# Association of four lipid-derived indicators with the risk of developing type 2 diabetes: a Chinese population-based cohort study

**DOI:** 10.1186/s12944-023-01790-7

**Published:** 2023-02-14

**Authors:** Linfeng He, Wenbin Zheng, Zeyu Li, Wen Kong, Tianshu Zeng

**Affiliations:** 1 Department of Endocrinology, Union Hospital, Tongji Medical College, Huazhong University of Science and Technology, Wuhan, Hubei China; 2 Hubei Provincial Clinical Research Center for Diabetes and Metabolic Disorders, Huazhong University of Science and Technology, Wuhan, Hubei China; 3 Hubei Key Laboratory of Metabolic Abnormalities and Vascular Aging, Huazhong University of Science and Technology, Wuhan, Hubei China

**Keywords:** Type 2 diabetes, Lipid ratio, Triglyceride glucose index, Prediction

## Abstract

**Background:**

Studies have reported that lipid-derived indicators are associated with type 2 diabetes (T2D) in various populations; however, it is unclear which lipid-derived indicators could effectively predict T2D risk. Therefore, this study aimed to explore the association between four lipid-derived indicators and T2D risk.

**Methods:**

This was a post-hoc analysis from a large cohort that included data from 114,700 Chinese individuals aged 20 years and older from 11 cities and 32 sites. The association between four lipid-derived indicators and T2D risk was determined using Kaplan-Meier (KM) survival curves, Cox regression, and restricted cubic spline analyses. This study used receiver operating characteristic (ROC) curves for assessing the ability of four lipid-derived indicators to accurately predict the development of T2D during follow-up.

**Results:**

This study included a total of 114,700 participants, with a mean age of 44.15. These individuals were followed up for 3.1 years, of which 2668 participants developed T2D. ROC curve analysis showed that TyG was the most robust predictor of 3-year [aera under the ROC (AUC) = 0.77, 95% CI: 0.768, 0.772] and 5-year T2D risk (AUC = 0.763, 95% CI: 0.760, 0.765). In addition, sensitivity analysis showed an association between TyG and an increased incidence of T2D.

**Conclusions:**

The results suggest that TyG was a superior for predicting the risk of developing T2D in the general Chinese population.

**Supplementary Information:**

The online version contains supplementary material available at 10.1186/s12944-023-01790-7.

## Introduction

Type 2 diabetes (T2D) is a chronic disease characterized by hyperglycemia and increases the risk of developing cardiovascular diseases [1–3], leading to loss of disability-adjusted life expectancy loss and death [4]. Recently, several developing countries have reported an increasing trend in the incidence. Currently, China accounts for the most cases of T2D globally owing to its vast population and elevated standard of living [5]. Hence, prevention and early management of T2D have become a major task for Chinese society.

Lipid metabolism disorders cause and manifest in diabetes [6, 7]. High triglyceride (TG) and decreased high-density lipoprotein (HDL) are indicators of lipid metabolism disorders in T2D [7]. Furthermore, substantial evidence has shown [8–12] an association between lipid levels and T2D risk, and changes in lipid levels precede hyperglycemia [8, 13]. In brief, an association was observed between alterations in lipid levels and the risk of developing T2D in the future. Several parameters correlated with T2D, like triglyceride glucose index (TyG), TG/HDL ratio, non-HDL/HDL ratio, and low-density lipoprotein cholesterol/HDL ratio are frequently used to determine T2D risk and are superior to single lipid indicators in predicting T2D risk [14–17]. However, it is unclear which lipid-derived indicator could accurately predict T2D risk in the Chinese population. Therefore, this study analyzed the association between four lipid-derived indicators and T2D risk in 114,700 individuals from multiple centers in China. This study aimed to identify the lipid-derived indicators that could accurately predict the risk of developing T2D in clinical settings.

## Methods

### Study population

**Fig. 1:**
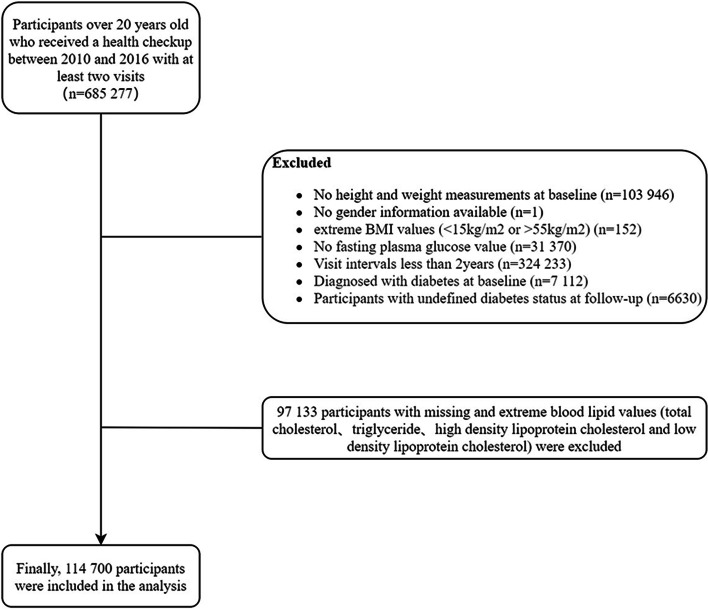
Flow chart of study subjects

A total of 685,277 Chinese individuals aged ≥20 years were recruited by the Rich Medical Group from 32 sites and 11 cities (Beijing, Changzhou, Chengdu, Guangzhou, Hefei, Nanjing, Nantong, Shanghai, Shenzhen, Suzhou, and Wuhan) in China between 2010 and 2016 [18], with at least two visits. The present study conducted a post hoc analysis of this cohort. The individuals with missing data on lipid markers were excluded from the study. Finally, a total of 114,700 individuals were eventually included in this study. Figure 1 shows the study selection process.

### Data collection

The demographic information such as the participant’s age, gender, weight, height, blood pressure readings (both systolic and diastolic), fasting plasma glucose (FPG), TG, LDL, HDL, total cholesterol (TC), smoking habits, alcohol use, and family history of diabetes were collected. The data were retrieved from the electronic medical record system. The formula was used to calculate the indices was as follows: body mass index (BMI): weight (kg)/height (m2), non-HDL/HDL = (TC-HDL)/HDL, and TyG = Ln(FPG TG/2).

### Diagnosis of T2D

Participants were followed-up for a mean duration of 3.1 ± 0.9 years. T2D was defined as FPG 126 mg/dL or self-reported T2D during follow-up.

### Statistical analysis

This study used student’s t-test, chi-squared, and rank-sum tests to determine differences between groups. Cox regression analysis was conducted for calculating the risk factor and hazard ratios (HRs) were calculated. Kaplan–Meier (KM) curves were used to analyze the risk of developing T2D over time for four lipid-derived indicators with a log-rank test. This study constructed ROC curves to evaluate the ability of four lipid-derived indicators in predicting T2D risk in participants. This study performed sensitivity analyses by constructing different regression models and stratified analyses for determining the stability of the results. EmpowerStats (www.empowerstats.com) and R package (http://www.r-project.org) were used for data analysis. *P* <  0.05 was considered statistically significant.

## Results

### Study population characteristics

**Fig. 2:**
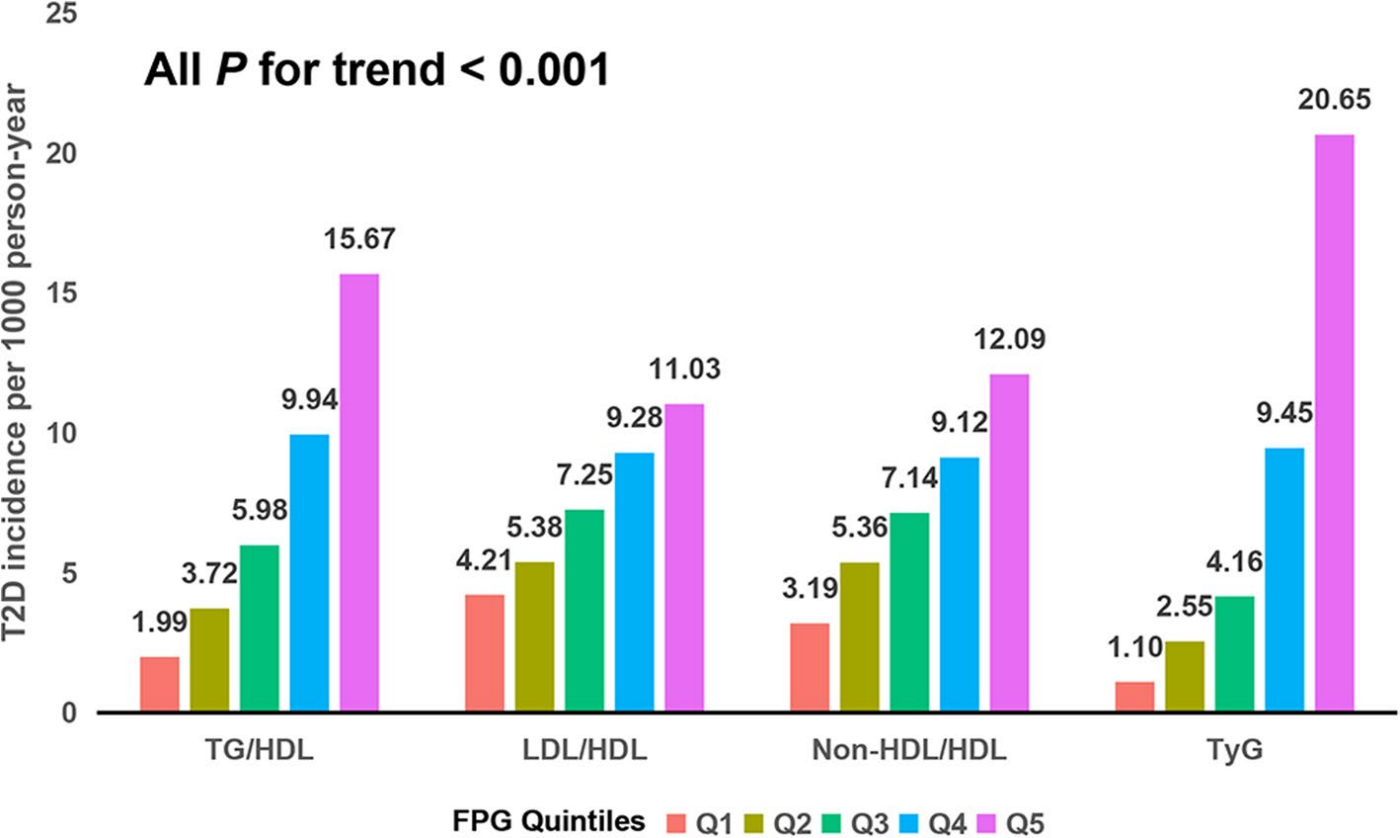
Association of four lipid-derived indicators with the incidence of T2D

This post hoc analysis included 114,700 participants with a mean age of 44.15 years, of which 62,093 participants were males (54.14%) and 52,067 participants were females. The participants were followed-up time of 3.1 ± 0.9 years, and 1877 males and 791 females were diagnosed with T2D. Newly diagnosed patients with T2D had significantly higher baseline parameters compared to participants without diabetes (Table 1). In addition, a positive correlation of lipid-derived indicators increases with the level of T2D (Fig. 2).

**Table 1:** Baseline characteristics of the study population

	No diabetes	New-onset diabetes	***P***-value
N	112,032	2668	< 0.001
Age (years)	43.85 ± 12.80	56.65 ± 12.65	< 0.001
Gender			< 0.001
Female	51,816 (46.25)	791 (29.65)	
Male	60,216 (53.75)	1877 (70.35)	
Height (cm)	166.32 ± 8.32	166.52 ± 8.55	0.221
Weight (kg)	64.80 ± 12.03	72.55 ± 13.07	< 0.001
BMI (kg/m^2^)	23.31 ± 3.27	26.04 ± 3.43	< 0.001
Systolic blood pressure (mmHg)	119.22 ± 16.52	132.00 ± 18.78	< 0.001
Diastolic blood pressure (mmHg)	74.36 ± 10.91	80.58 ± 11.93	< 0.001
FPG (mg/dL)	88.67 ± 10.50	106.56 ± 12.72	< 0.001
TC (mg/dL)	185.48 ± 34.54	196.41 ± 36.47	< 0.001
TG (mg/dL)	97.46 (68.22, 146.20)	151.50 (104.50, 221.50)	< 0.001
HDL (mg/dL)	53.02 ± 11.54	49.82 ± 11.07	< 0.001
LDL (mg/dL)	107.14 ± 26.26	112.32 ± 27.16	< 0.001
Alanine aminotransferase (U/L)	18.00 (13.00, 27.20)	25.10 (18.00, 39.45)	< 0.001
Blood urea nitrogen (mmol/L)	4.68 ± 1.17	5.02 ± 1.29	< 0.001
Serum creatinine (μmol/L)	70.31 ± 15.82	73.02 ± 16.49	< 0.001
Smoking status			< 0.001
Never smoker	23,767 (21.21)	386 (14.47)	
Ever smoker	1272 (1.14)	46 (1.72)	
Current smoker	6372 (5.69)	256 (9.6)	
Unknown	80,621 (71.96)	1980 (74.21)	
Drinking status			0.002
Never drinker	25,227 (22.52)	541 (20.28)	
Ever drinker	5353 (4.78)	116 (4.35)	
Current drinker	831 (0.74)	31 (1.16)	
Unknown	80,621 (71.96)	1980 (74.21)	
Family history of diabetes			< 0.001
No	109,547 (97.78)	2570 (96.33)	
Yes	2485 (2.22)	98 (3.67)	

Data are expressed as mean ± SD, median (IQR) and n (%)

*BMI* body mass index, *FPG* fasting plasma glucose, *TC* total cholesterol, *TG* triglyceride, *LDL* low-density lipoprotein, *HDL* high-density lipoprotein

### Univariate analysis

**Fig. 3:**
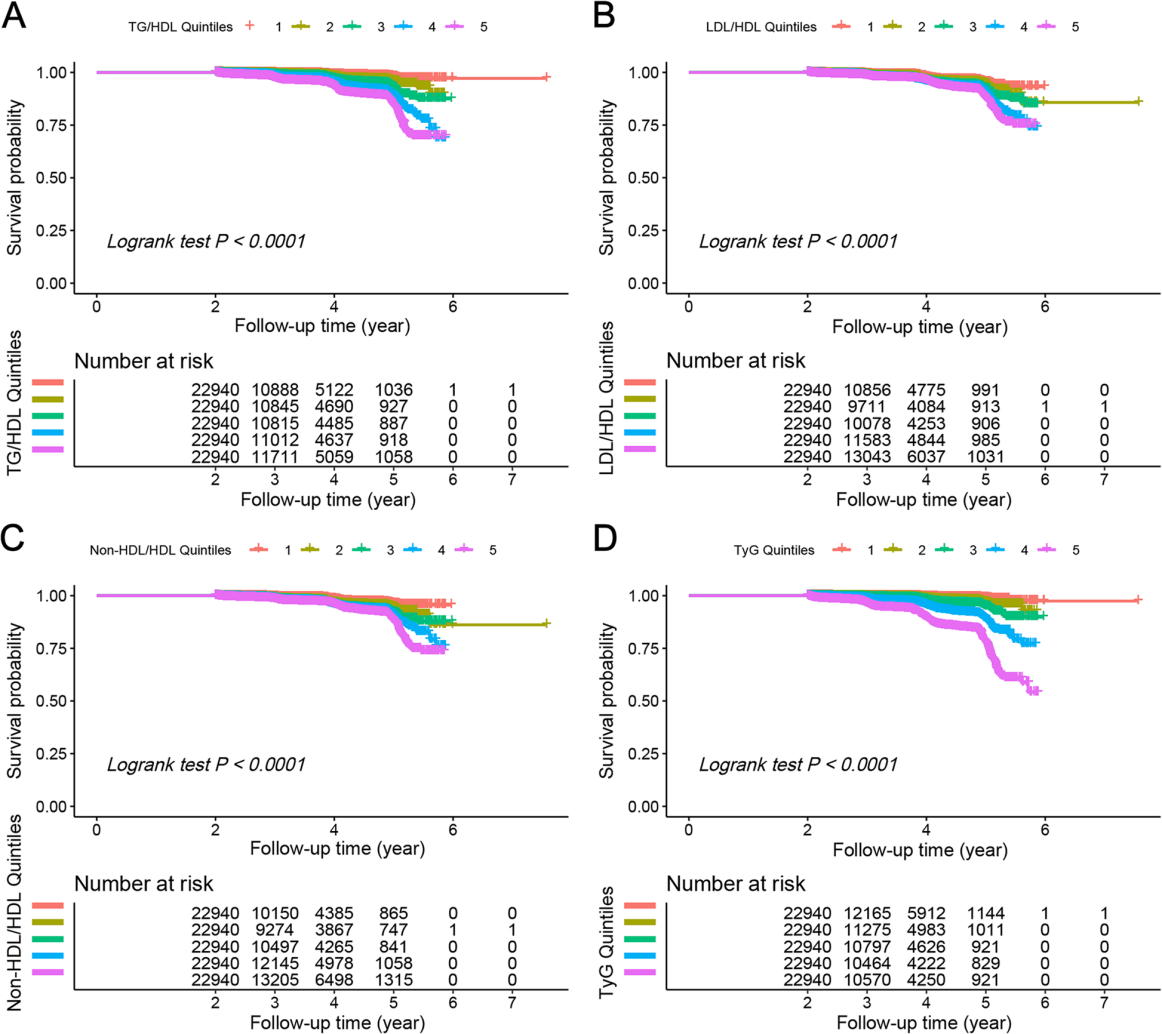
Kaplan-Meier analysis of four lipid-derived indicators and T2D. **A** TG/HDL, **B** LDL/HDL, **C** Non-HDL/HDL, **D** TyG

All baseline indicator was positively correlated with T2D risk. A negative correlation was observed only between HDL and T2D risk. Males, participants with smoking and alcohol consumption habits, and those with a family history of diabetes were more likely to develop T2D (Supplementary Table 1). Figure 3 shows the KM survival curve of the incidence of T2D in participants with different levels of the four lipid-derived indicators over time. Higher lipid derivatives indicated a higher risk of developing T2D (*P* <  0.001 for all log-rank tests).

### Lipid-derived indicators for predicting T2D risk

**Fig. 4:**
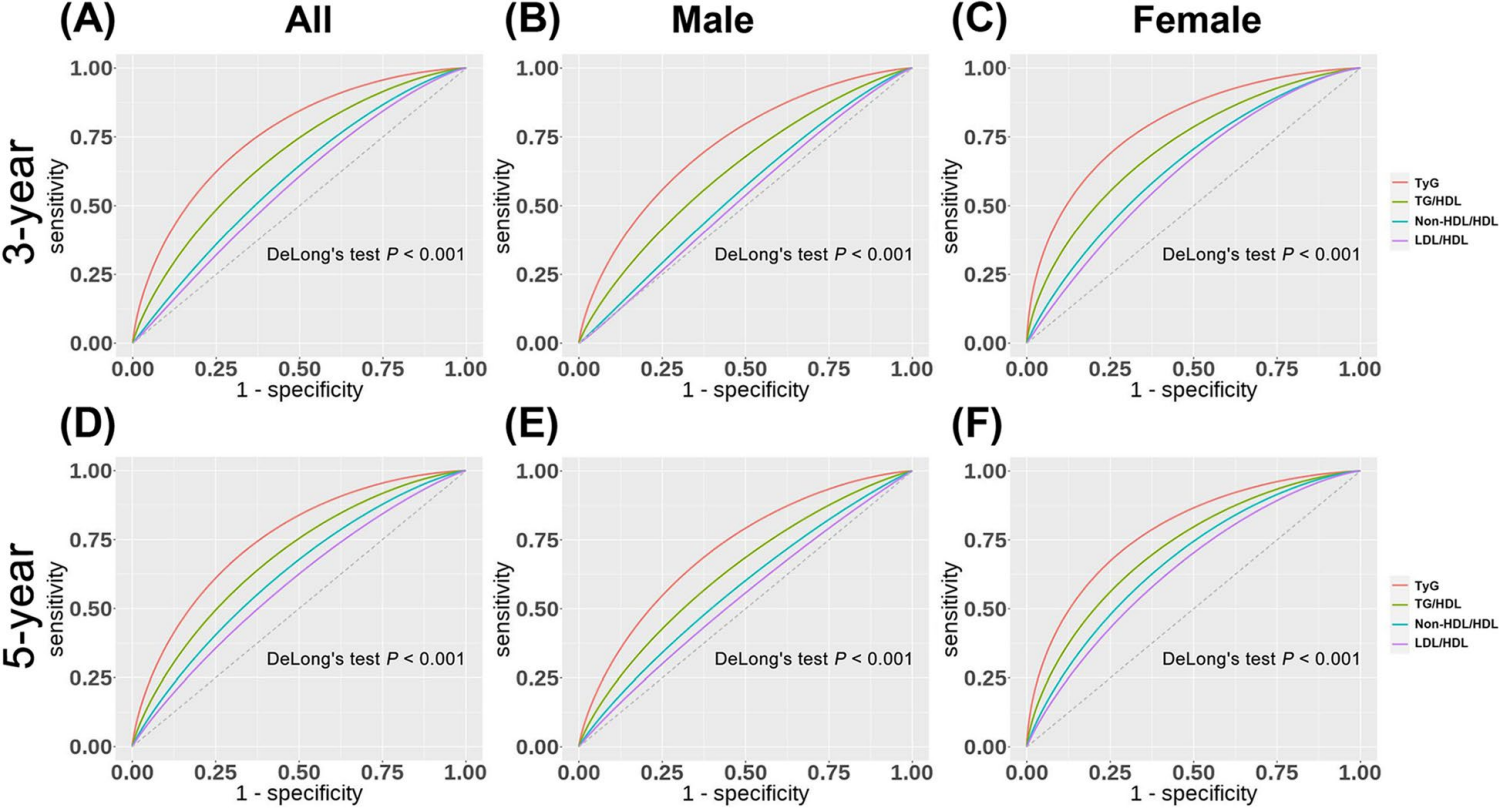
ROC curve analysis of the predictive efficacy of four lipid-derived indicators. **A**, **B** and **C** are ROC curves predicting the 3-year risk of T2D for all, male and female populations. **D**, **E** and **F** are ROC curves predicting the 5-year risk of T2D for all, male and female populations

The Harrell’s concordance index (C-index) showed that TyG could better predict T2D risk (C-index for all participants: 0.769, male: 0.724, female: 0.81; Supplementary Table 3). The effectiveness of the four lipid-derived indicators in predicting T2D at 3 and 5 years was ranked as follows: TyG > TG/HDL > non-HDL/HDL > LDL/HDL in all participants irrespective of their gender (all Delong’s test *P* <  0.001; Fig. 4, Supplementary Tables 2 and 4). The cutting-off values (highest value for Youden index) of TyG, TG/HDL, non-HDL/HDL, and LDL/HDL for predicting the 3-year risk of T2D in all participants were 8.57, 1.13, 2.49, and 1.91, respectively, and for the 5-year risk of T2D were 8.57, 1.02, 2.46, and 1.99 respectively.

### Association between lipid-derived indicators and T2D

**Fig. 5:**
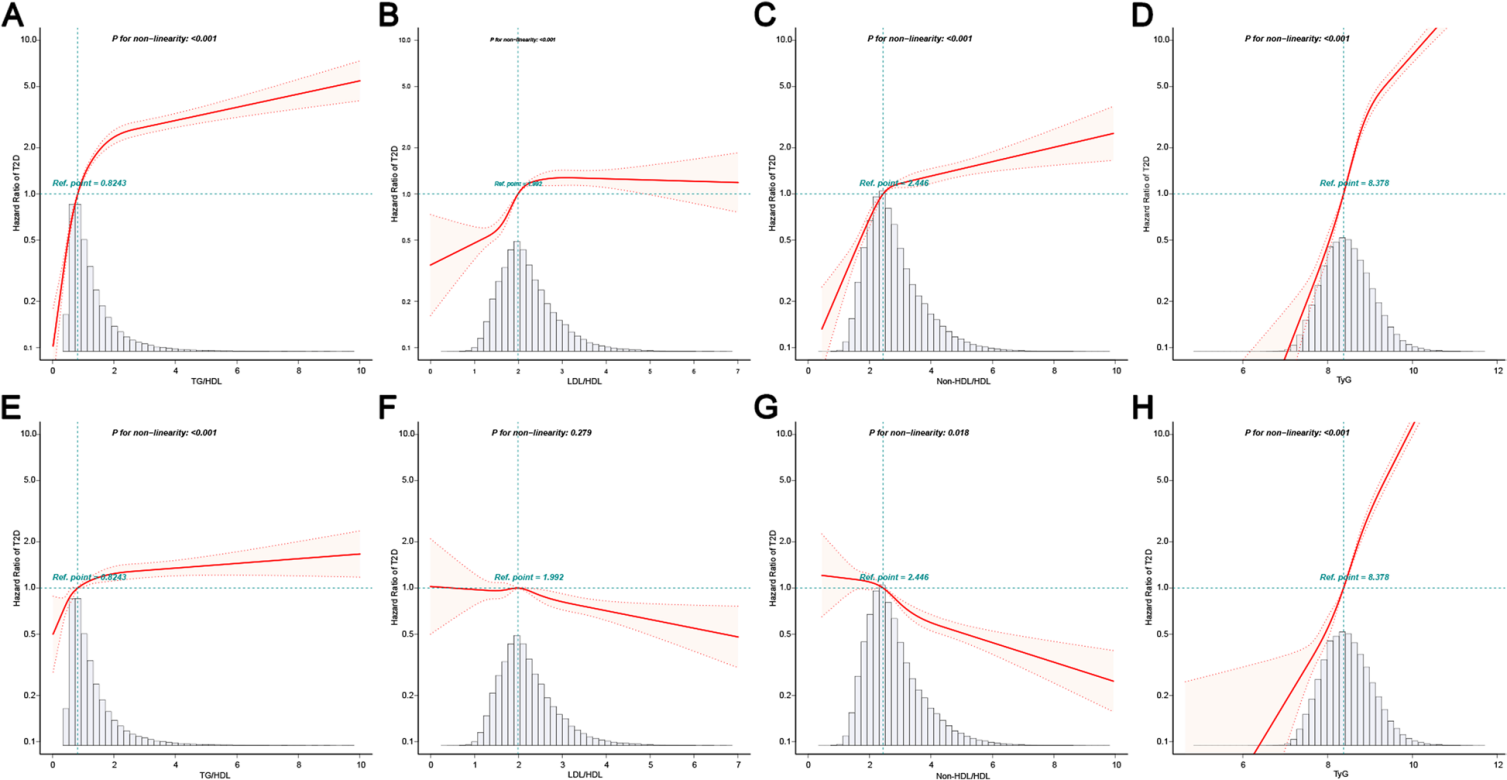
Five knots of restriction cubic spline (RCS) plots of four lipid-derived indicators with T2D. Unadjusted RCS: **A** TG/HDL, **B** LDL/HDL, **C** Non-HDL/HDL, **D** TyG; Multivariate-adjusted RCS: **E** TG/HDL, **F** LDL/HDL, **G** Non-HDL/HDL, **H** TyG

The multivariate-adjusted HRs and 95% confidence intervals (CIs) for the four lipid-derived indicators with T2D risk were as follows: TG/HDL = 1.08 (1.05, 1.11), LDL/HDL = 0.88 (0.83, 0.93), non-HDL/HDL = 0.78 (0.74, 0.81) and TyG = 4.36 (4.05, 4.69) (Table 2). A strong association between TyG and T2D, with LDL/HDL and non-HDL/HDL risk was observed. The results of the multivariate-adjusted and univariate analyses performed on LDL/HDL and non-HDL/HDL were contradictory. Restricted cubic splines (RCS) analysis was performed to determine the association between four lipid-derived indicators and T2D. The unadjusted and full model multivariate-adjusted RCS results were almost similar to the Cox regression results (Fig. 5).

**Table 2:** Cox regression analysis of the relationship between four lipid-derived indicators and T2D

	HR (95% CI)		
	Crude model	Minimally model	Fully model
**TG/HDL**	1.24 (1.23, 1.26)	1.22 (1.20, 1.24)	1.08 (1.05, 1.11)
**TG/HDL Quintiles**			
Q1	Reference	Reference	Reference
Q2	1.94 (1.58, 2.38)	1.57 (1.28, 1.93)	1.29 (1.05, 1.59)
Q3	3.16 (2.61, 3.82)	2.14 (1.76, 2.59)	1.36 (1.12, 1.65)
Q4	5.20 (4.35, 6.23)	3.16 (2.63, 3.80)	1.64 (1.36, 1.98)
Q5	7.81 (6.56, 9.29)	4.49 (3.76, 5.37)	1.76 (1.46, 2.12)
*P* for trend	< 0.001	< 0.001	< 0.001
**LDL/HDL**	1.26 (1.21, 1.30)	1.11 (1.06, 1.16)	0.88 (0.83, 0.93)
**LDL/HDL Quintiles**			
Q1	Reference	Reference	Reference
Q2	1.38 (1.19, 1.61)	1.20 (1.03, 1.40)	0.99 (0.85, 1.16)
Q3	1.83 (1.58, 2.11)	1.40 (1.21, 1.61)	1.00 (0.86, 1.15)
Q4	2.19 (1.91, 2.51)	1.46 (1.27, 1.68)	0.92 (0.80, 1.07)
Q5	2.39 (2.09, 2.73)	1.47 (1.29, 1.68)	0.84 (0.72, 0.97)
*P* for trend	< 0.001	< 0.001	0.004
**Non-HDL/HDL**	1.18 (1.16, 1.20)	1.10 (1.07, 1.13)	0.78 (0.74, 0.81)
**Non-HDL/HDL Quintiles**			
Q1	Reference	Reference	Reference
Q2	1.81 (1.53, 2.14)	1.51 (1.28, 1.78)	0.98 (0.82, 1.16)
Q3	2.28 (1.94, 2.67)	1.64 (1.40, 1.92)	0.84 (0.71, 0.99)
Q4	2.64 (2.27, 3.07)	1.67 (1.43, 1.94)	0.71 (0.61, 0.84)
Q5	3.15 (2.72, 3.64)	1.76 (1.52, 2.04)	0.52 (0.44, 0.62)
*P* for trend	< 0.001	< 0.001	< 0.001
**TyG**	3.83 (3.64, 4.03)	3.22 (3.04, 3.41)	4.36 (4.05, 4.69)
**TyG Quintiles**			
Q1	Reference	Reference	Reference
Q2	2.48 (1.91, 3.23)	1.93 (1.49, 2.51)	1.83 (1.41, 2.39)
Q3	4.19 (3.28, 5.36)	2.74 (2.14, 3.52)	2.55 (1.99, 3.28)
Q4	9.93 (7.88, 12.51)	5.69 (4.50, 7.18)	5.44 (4.28, 6.92)
Q5	21.39 (17.10, 26.75)	11.29 (8.98, 14.18)	11.50 (9.03, 14.65)
*P* for trend	< 0.001	< 0.001	< 0.001

Crude model: not adjusted

Minimally model: adjusted for age and gender

Fully model: adjusted for minimally model, BMI, systolic blood pressure, diastolic blood pressure, alanine aminotransferase, blood urea nitrogen, serum creatinine, smoking status, drinking status and family history of diabetes. TG/HDL adjusted fully model + FPG + TC + LDL; LDL/HDL adjusted fully model + FPG + TG + TC; Non-HDL/HDL adjusted fully model + FPG + TG + TC+ LDL; TyG adjusted fully model + TC + LDL + HDL

*BMI* body mass index, *TyG* triglyceride glucose index, *FPG* fasting plasma glucose, *TC* total cholesterol, *TG* triglyceride, *LDL* low-density lipoprotein, *HDL* high-density lipoprotein, *HR* hazard ratio, *CI* Confidence interval

### Sensitivity analysis

This study performed a sensitivity analysis to determine the robustness of the association between the four lipid-derived indicators and T2D across different populations or subgroups. A significant reversal in the association between LDL/HDL and T2D was observed after adjustment for BMI, FPG, or TG (all *P*_interactions_ <  0.05). A negative association was observed between Non-HDL/HDL and T2D after further adjustment for BMI, FPG, or TG, and non-HDL/HDL interacted with BMI, FPG and TG (all *P*_interactions_ <  0.05). A steady association was observed between TyG, TG/HDL, and T2D (Supplementary Tables 5 and 6).

## Discussion

This study assessed the ability of four lipid-derived indicators (TG/HDL, LDL/HDL, non-HDL/HDL, TyG) for predicting T2D risk. In this study of a post hoc analysis of a multicenter Chinese population-based cohort study, high TG/HDL and TyG were significantly associated with an elevated risk of developing T2D. Time-dependent ROC curves analysis showed that TyG was the best predictor of 3- and 5-year T2D risk, with AUC values greater than 0.7. FPG, BMI, and TG mediated LDL/HDL and non-HDL/HDL to T2D.

Several studies have used TyG has been employed as a potential biomarker for insulin resistance (IR) [19–21] and have demonstrated superiority over the homeostatic model assessment of IR (HOMA-IR) [21]. This study revealed that TyG was associated with a higher incidence of T2D, consistent with the results of the previous study. Furthermore, a study on 5706 individuals from a rural China [22] showed that the risk of developing T2D increase with accumulated TyG. The optimal thresholds for predicting the risk of T2D in males and females were 8.76 and 8.64, respectively, consistent with this study. Similarly, Fu et al. showed that TyG could better predict T2D risk compared to TG/HDL in the older Chinese population [23], thereby supporting this study.

TG/HDL comprises TG and HDL. Studies have shown that high TG/HDL, TG, and low HDL accelerate T2D onset and progression [24–29]. This study further confirms the association between high TG/HDL levels and T2D risk. The optimal cut-off values of TG/HDL for predicting the 3- and 5-year risk of T2D, the ideal cut-off values for TG/HDL were 1.13 and 1.02, respectively, and the AUC values were 0.691 and 0.694, respectively. However, the efficacy of TG/HDL for predicting T2D risk was lower compared to TyG. These results are consistent with the study of Yang et al. [14] on Chinese patients with coronary heart disease and the study of Liu et al. [15] on the elderly Chinese population.

However, an association between LDL/HDL, non-HDL/HDL, and T2D risk reported by us differs from previous studies. Few studies have evaluated the correlation between LDL/HDL and T2D risk. Wei et al. [16] were the first to demonstrate a correlation between cumulative LDL/HDL and T2D risk in the Chinese population. The difference in results was observed when the multivariate Cox regression was not adjusted for HDL; however, the results were consistent with their study after further adjustment. HDL is a component of LDL/HDL; hence it would be more appropriate to make no adjustments for HDL. Shen et al. [17] showed a correlation between non-HDL/HDL and T2D risk in 15,464 Japanese individuals above 18 years. Similar results were obtained in a study by Zhang et al. [30], with 4882 Chinese individuals above 40 years. Unlike previous studies, the present study shows a positive association between non-HDL/HDL and T2D risk only in participants with lower FPG and TG levels or in participants who were not obese after adjusting for confounders. Based on this study and the available literature, the possible explanations for the association between the four lipid-derived indicators and T2D are as follows. High TG and low HDL are important features of IR, which is often the predecessor to prediabetes [31]. High TG levels attenuate insulin sensitivity in peripheral tissues [32]. Simultaneously, a decrease in adenylate-activated protein kinase activity promotes TG accumulation, thereby leading to a shift in insulin signaling and glucagon secretion by pancreatic α-cells [33]. Together, this promotes a vicious cycle between TG and IR. On the contrary, HDL enhances insulin resistance and protects against cytokines- or high-glucose-induced apoptosis pancreatic β-cell apoptosis via apolipoprotein A1 (ApoA1) [34–36]. Further, an association was observed between TyG, TG/HDL levels, and T2D risk. Several studies have shown LDL as a risk factor for cardiovascular disease and mediate its destructive effects via oxidative stress [37–39]. .Further, an association exists between LDL and diabetes. LDL inhibits insulin secretion [34], and oxidized LDL reduces insulin secretion and induces cell death by activating the c-Jun terminal kinase pathway [40]. Non-HDL, which includes LDL, plays a similar role [30]. Previous studies have shown a positive correlation between LDL/HDL, non-HDL/HDL, and T2D risk.

Nevertheless, this study showed a positive correlation between LDL/HDL, non-HDL/HDL, and T2D risk only in participants with low FPG and participants who were not obese. The physical examination showed that the population was mostly healthy, with LDL levels within the normal range (< 120 mg/dL) [41]; hence this study cautiously speculates that the effect of LDL on islet function was insignificant. Moreover, previous studies have shown a significant association between LDL and diabetes in the Western population, whereas high TG levels were observed in the Chinese population [42]. In this study, the effect of obesity and high glucose level exceeded the effects of LDL and non-HDL on T2D risk in the Chinese population. Since LDL/HDL and non-HDL/HDL are not universal, TyG and TG/HDL could be better indicators of T2D risk in the Chinese population. However, the specific underlying mechanisms and links require further investigation.

With the advancement in people’s understanding of diabetes, new treatment strategies are constantly emerging. Sodium-glucose cotransporter 2 inhibitors can control blood glucose levels in the blood, reduce the risk of cardiovascular disease, and improve the prognosis of patients with diabetes [43–45]. These advancements have shed new light on the treatment of patients with diabetes. However, the prevention of diabetes still remains the key. This study revealed that TyG and TG/HDL were reliable predictors of lipid-derived predictors of T2D risk in the general Chinese population. Of which, TyG was the best predictor of T2D risk. Given the high prevalence and increasing trend of T2D in the Chinese population [5], determining TyG levels in individuals could aid in preventing T2D. TyG is an alternate marker for insulin resistance and a promising indicator for predicting the early risk of developing T2D. Future more well-designed cohort studies are also encouraged to validate the results.

### Comparisons with other studies and what does the current work add to the existing knowledge

Previous studies have demonstrated the association between lipids and T2D and the effectiveness of four lipid derivatives in predicting T2D risk [8–12]. However, the identification of the lipid derivative for predicting T2D risk in a clinical setting is still unclear. Hence, this study analyzed the association between four common lipid derivatives and T2D in the general Chinese population and identified the best lipid-derived indicators for predicting T2D risk. These results support the selection of TyG as an indicator for predicting T2D risk in clinical settings.

### Study strengths and limitations

Based on the available literature, this study has determined the association between four lipid-derived indicators (TG/HDL, LDL/HDL, non-HDL/HDL, and TyG) with the risk of developing T2D for the first time. Second, this multicenter cohort study included 114,700 Chinese individuals. Thus, our results could more accurately reflect the status of T2D risk in the Chinese population. Nevertheless, this study has a few limitations: (1) Post hoc analysis of retrospective studies could be limited by the participant’s review records. (2) This study only included patients with data on FPG and self-reported criteria to diagnose diabetes, and OGTT and patients with glycated hemoglobin-diagnosed diabetes could be missed. In addition, self-reported diabetes cases could be biased due to the lack of experience of the investigators and awareness among the patients. (3) Furthermore, this study could not differentiate between patients with type 1 diabetes (T1D) and T2D due to the lack of diabetes-related antibodies. (4) It is important to note that these results were only observed in the Chinese population; it is unclear if these results could be extrapolated to other ethnic groups.

## Conclusions

This study demonstrated that TyG has the highest predictive efficacy in predicting the risk of T2D compared to the other three lipid-derived indicators (TG/HDL, non-HDL/HDL, and LDL/HDL) in the general Chinese population. In clinical practice, TyG is a superior lipid-derived indicator for identifying individuals at high risk for T2D in clinical settings.

## Supplementary Information

**Additional file 1: Supplementary Table 1.** Univariate analysis of T2D. **Supplementary Table 2.** Predictive efficacy of four lipid-derived indicators for T2D. **Supplementary Table 3.** C-index for four lipid-derived indicators. **Supplementary Table 4.** Comparison of the AUC values of four lipid-derived indicators. **Supplementary Table 5.** Cox regression analysis of predictors associated with new-onset T2DM. **Supplementary Table 6** Subgroup analysis of four lipid-derived indicators.

## Data Availability

The data used in this study can be obtained from the ‘DATADRYAD’ database (www.Datadryad.org).

## Abbreviations

BMI: Body mass index
FPG: Fasting plasma glucose
TC: Total cholesterol
TG: Triglyceride
LDL: Low-density lipoprotein cholesterol
HDL: High-density lipoprotein cholesterol
TyG: Triglyceride glucose index
TG/HDL: Triglyceride to high-density lipoprotein cholesterol ratio
non-HDL/HDL: Non-high-density lipoprotein cholesterol to high-density lipoprotein cholesterol ratio
LDL/HDL: Low-density lipoprotein cholesterol to high-density lipoprotein cholesterol ratio
ApoA1: Apolipoprotein A1
OGTT: Oral Glucose Tolerance Test
IR: Insulin resistance
HOMA-IR: Homeostatic model assessment of IR
T2D: Type 2 diabetes
T1D: Type 1 diabetes
C-index Harrell’s: Concordance index
ROC: Receiver operating characteristic
AUC: Area under the ROC
HR: Hazard ratio
CI: Confidence interval
