# Supplementary material for: Association of four lipid-derived indicators with the risk of developing type 2 diabetes: a Chinese population-based cohort study
Source: Lipids Health Dis. 2023 Feb 14;22:24. doi: 10.1186/s12944-023-01790-7 (PMC9930254; doi:10.1186/s12944-023-01790-7)
Supplement: Supplementary file 1 — Additional file 1: Supplementary Table 1. Univariate analysis of T2D. Supplementary Table 2. Predictive efficacy of four lipid-derived indicators for T2D. Supplementary Table 3. C-index for four lipid-derived indicators. Supplementary Table 4. Comparison of the AUC values of four lipid-derived indicators. Supplementary Table 5. Cox regression analysis of predictors associated with new-onset T2DM. Supplementary Table 6 Subgroup analysis of four lipid-derived indicators. [file 12944_2023_1790_MOESM1_ESM.docx]

| **Supplementary Table 1 Univariate analysis of T2D** | | | |
| --- | --- | --- | --- |
|  | **Statistics** | **HR (95% CI)** | ***P-*value** |
| Age | 44.15 ± 12.94 | 1.06 (1.06, 1.66) | < 0.001 |
| Gender |  |  | < 0.001 |
| Female | 52607 (45.86%) | Reference |  |
| Male | 62093 (54.14%) | 1.98 (1.82, 2.15) | < 0.001 |
| BMI | 23.38 ± 3.30 | 1.22 (1.21, 1.23) | < 0.001 |
| Systolic blood pressure | 119.52 ± 16.69 | 1.04 (1.04, 1.04) | < 0.001 |
| Diastolic blood pressure | 74.50 ± 10.98 | 1.04 (1.04, 1.05) | < 0.001 |
| TG/HDL | 0.82 (0.53-1.35) | 1.24 (1.23, 1.26) | < 0.001 |
| LDL/HDL | 2.12 ± 0.71 | 1.26 (1.21, 1.30) | < 0.001 |
| Non-HDL/HDL | 2.65 ± 1.00 | 1.18 (1.16, 1.20) | < 0.001 |
| TyG | 8.42 ± 0.61 | 3.83 (3.64, 4.03) | < 0.001 |
| FPG | 89.09 ± 10.90 | 1.14 (1.13, 1.14) | < 0.001 |
| TC | 185.74 ± 34.62 | 1.01 (1.01, 1.01) | < 0.001 |
| TG | 98.35 (68.22-148.85) | 1.00 (1.00, 1.00) | < 0.001 |
| HDL | 52.94 ± 11.54 | 0.99 (0.98, 0.99) | < 0.001 |
| LDL | 107.26 ± 26.29 | 1.01 (1.01, 1.01) | < 0.001 |
| [Alanine aminotransferase](file:///D:\%25E5%25AE%2589%25E8%25A3%2585%25E7%25A8%258B%25E5%25BA%258F\Youdao\Dict\9.0.4.0\resultui\html\index.html#\javascript:;) | 18.10 (13.00-27.60) | 1.00 (1.00, 1.00) | < 0.001 |
| Blood urea nitrogen | 4.69 ± 1.18 | 1.21 (1.18, 1.24) | < 0.001 |
| Serum creatinine | 70.37 ± 15.84 | 1.01 (1.01, 1.01) | < 0.001 |
| Smoking status |  |  | < 0.001 |
| Never smoker | 24153 (21.06%) | Reference |  |
| Ever smoker | 1318 (1.15%) | 2.04 (1.50, 2.76) | < 0.001 |
| Current smoker | 6628 (5.78%) | 2.36 (2.02, 2.76) | < 0.001 |
| Unkonwn | 82601 (72.01%) | 1.55 (1.39, 1.72) | < 0.001 |
| Drinking status |  |  | < 0.001 |
| Never drinker | 25768 (22.47%) | Reference |  |
| Ever drinker | 5469 (4.77%) | 0.94 (0.77, 1.14) | 0.51 |
| Current drinker | 862 (0.75%) | 1.96 (1.37, 2.82) | < 0.001 |
| Unkonwn | 82601 (72.01%) | 1.73 (1.07, 1.29) | < 0.001 |
| Family history of diabetes |  |  | 0.002 |
| No | 112117 (97.75%) | Reference |  |
| Yes | 2583 (2.25%) | 1.40 (1.15, 1.72) | 0.001 |
| TyG triglyceride glucose index, FPG fasting plasma glucose, TC total cholesterol, TG triglyceride, LDL low density lipoprotein, HDL High density lipoprotein, HR hazard ratio, CI Confidence interval | | | |

| **Supplementary Table 2 Predictive efficacy of four lipid-derived indicators for T2D** | | | | | | | | |
| --- | --- | --- | --- | --- | --- | --- | --- | --- |
|  | **AUC** | **95%CI** | **Cut-off value** | **Sensitivity,%** | **Specificity,%** | **PPV,%** | **NPV,%** | **Youden index** |
| **3-year** |  |  |  |  |  |  |  |  |
| **All** |  |  |  |  |  |  |  |  |
| TyG | 0.77 | 0.768, 0.772 | 8.57 | 79.71 | 62.12 | 2.1 | 99.7 | 0.4183 |
| TG/HDL | 0.691 | 0.688, 0.693 | 1.13 | 62.06 | 67.25 | 1.8 | 99.5 | 0.2931 |
| Non-HDL/HDL | 0.608 | 0.605, 0.611 | 2.49 | 64.97 | 52.26 | 1.3 | 99.4 | 0.1723 |
| LDL/HDL | 0.59 | 0.587, 0.593 | 1.91 | 71.43 | 44.43 | 1.2 | 99.4 | 0.1586 |
| **Male** |  |  |  |  |  |  |  |  |
| TyG | 0.726 | 0.722, 0.729 | 8.84 | 66.33 | 67.83 | 2.6 | 99.4 | 0.3416 |
| TG/HDL | 0.635 | 0.631, 0.638 | 1.13 | 67.09 | 54.55 | 1.9 | 99.2 | 0.2164 |
| Non-HDL/HDL | 0.549 | 0.545, 0.553 | 2.49 | 66.58 | 41.67 | 1.4 | 99 | 0.0825 |
| LDL/HDL | 0.535 | 0.531, 0.539 | 1.91 | 73.21 | 34.71 | 1.4 | 99 | 0.0792 |
| **Female** |  |  |  |  |  |  |  |  |
| TyG | 0.81 | 0.806, 0.813 | 8.5 | 78.41 | 72.53 | 1.7 | 99.8 | 0.5094 |
| TG/HDL | 0.733 | 0.729, 0.737 | 0.91 | 63.49 | 72.58 | 1.4 | 99.7 | 0.3607 |
| Non-HDL/HDL | 0.66 | 0.656, 0.664 | 2.37 | 66.98 | 59 | 1 | 99.7 | 0.2598 |
| LDL/HDL | 0.641 | 0.637, 0.645 | 2.04 | 59.68 | 64.94 | 1 | 99.6 | 0.2462 |
| **5-year** |  |  |  |  |  |  |  |  |
| **All** |  |  |  |  |  |  |  |  |
| TyG | 0.763 | 0.760, 0.765 | 8.57 | 77.81 | 62.61 | 4.5 | 99.2 | 0.4042 |
| TG/HDL | 0.694 | 0.691, 0.697 | 1.02 | 66.93 | 62.45 | 3.9 | 98.8 | 0.2938 |
| Non-HDL/HDL | 0.631 | 0.628, 0.633 | 2.46 | 69.19 | 51.02 | 3.1 | 98.7 | 0.2021 |
| LDL/HDL | 0.604 | 0.602, 0.607 | 1.99 | 67.17 | 50.09 | 2.9 | 98.5 | 0.1726 |
| **Male** |  |  |  |  |  |  |  |  |
| **Supplementary Table 2 continued** | | | | | | | | |
| TyG | 0.721 | 0.718, 0.725 | 8.78 | 68.71 | 64.41 | 5.3 | 98.6 | 0.3312 |
| TG/HDL | 0.642 | 0.638, 0.646 | 1.16 | 65.7 | 56.41 | 4.2 | 98.3 | 0.2211 |
| Non-HDL/HDL | 0.576 | 0.572, 0.580 | 2.49 | 69.28 | 41.97 | 3.4 | 97.9 | 0.1125 |
| LDL/HDL | 0.553 | 0.549, 0.557 | 2.36 | 45.6 | 63.18 | 3.5 | 97.5 | 0.0878 |
| **Female** |  |  |  |  |  |  |  |  |
| TyG | 0.798 | 0.794, 0.801 | 8.45 | 78.49 | 69.77 | 3.7 | 99.5 | 0.4826 |
| TG/HDL | 0.736 | 0.732, 0.739 | 0.93 | 62.19 | 74.16 | 3.4 | 99.3 | 0.3635 |
| Non-HDL/HDL | 0.684 | 0.680, 0.688 | 2.37 | 70.4 | 59.27 | 2.5 | 99.3 | 0.2967 |
| LDL/HDL | 0.656 | 0.652, 0.660 | 2.03 | 61.54 | 64.61 | 2.5 | 99.1 | 0.2615 |
| TyG triglyceride glucose index, FPG fasting plasma glucose, TC total cholesterol, TG triglyceride, LDL low density lipoprotein, HDL High density lipoprotein, area under the ROC, CI Confidence interval | | | | | | | | |

**Supplementary Table 3 C-index for four lipid-derived indicators**

|  | **C-index (95% CI)** | | |
| --- | --- | --- | --- |
|  | **All** | **Male** | **Female** |
| TyG | 0.769 (0.759, 0.779) | 0.724 (0.71, 0.738) | 0.81 (0.792, 0.828) |
| TG/HDL | 0.682 (0.668, 0.694) | 0.624 (0.608, 0.64) | 0.729 (0.707, 0.751) |
| Non-HDL/HDL | 0.585 (0.573, 0.594) | 0.525 (0.509, 0.541) | 0.641 (0.619, 0.663) |
| LDL/HDL | 0.572 (0.56, 0.584) | 0.517 (0.501, 0.533) | 0.625 (0.603, 0.647) |

TyG triglyceride glucose index, FPG fasting plasma glucose, TC total cholesterol,

TG triglyceride, LDL low density lipoprotein, HDL High density lipoprotein, C-index

Harrell's concordance index; CI Confidence interval

**Supplementary Table 4 Comparison of the AUC values of four lipid-derived indicators**

|  | **Difference between Area (95%CI) *P*** | | | | | |
| --- | --- | --- | --- | --- | --- | --- |
|  | **All** | | **Male** | | **Female** | |
| **3-year** |  |  |  |  |  |  |
| TyG *vs* TG/HDL | 0.0795 (0.0734, 0.0855) | < 0.001 | 0.0911 (0.0831, 0.099) | < 0.001 | 0.0769 (0.0656, 0.0881) | < 0.001 |
| TyG *vs* Non-HDL/HDL | 0.162 (0.147, 0.177) | < 0.001 | 0.177 (0.157, 0.196) | < 0.001 | 0.15 (0.123, 0.176) | < 0.001 |
| TyG *vs* LDL/HDL | 0.18 (0.163, 0.197) | < 0.001 | 0.19 (0.168, 0.213) | < 0.001 | 0.169 (0.139, 0.199) | < 0.001 |
| TG/HDL *vs* Non-HDL/HDL | 0.0826 (0.0689, 0.0963) | < 0.001 | 0.0855 (0.068, 0.103) | < 0.001 | 0.0729 (0.0477, 0.0982) | < 0.001 |
| TG/HDL *vs* LDL/HDL | 0.1 (0.0835, 0.117) | < 0.001 | 0.0992 (0.0776, 0.121) | < 0.001 | 0.0921 (0.0617, 0.123) | < 0.001 |
| Non-HDL/HDL *vs* LDL/HDL | 0.0178 (0.0102, 0.0255) | < 0.001 | 0.0136 (0.0041, 0.0231) | 0.005 | 0.0192 (0.0056, 0.0327) | 0.006 |
| **5-year** |  |  |  |  |  |  |
| TyG *vs* TG/HDL | 0.0686 (0.0645, 0.0727) | < 0.001 | 0.0795 (0.074, 0.085) | < 0.001 | 0.0621 (0.0548, 0.0694) | < 0.001 |
| TyG *vs* Non-HDL/HDL | 0.132 (0.122, 0.142) | < 0.001 | 0.145 (0.131, 0.159) | < 0.001 | 0.113 (0.0953, 0.131) | < 0.001 |
| TyG *vs* LDL/HDL | 0.158 (0.146, 0.17) | < 0.001 | 0.168 (0.153, 0.183) | < 0.001 | 0.142 (0.122, 0.163) | < 0.001 |
| TG/HDL *vs* Non-HDL/HDL | 0.0634 (0.0541, 0.0727) | < 0.001 | 0.0657 (0.0538, 0.0776) | < 0.001 | 0.0513 (0.0344, 0.0682) | < 0.001 |
| TG/HDL *vs* LDL/HDL | 0.0896 (0.0784, 0.101) | < 0.001 | 0.0886 (0.0743, 0.103) | < 0.001 | 0.08 (0.0599, 0.1) | < 0.001 |
| Non-HDL/HDL *vs* LDL/HDL | 0.0262 (0.0214, 0.0309) | < 0.001 | 0.0229 (0.0168, 0.029) | < 0.001 | 0.0287 (0.0208, 0.0366) | < 0.001 |

TyG triglyceride glucose index, FPG fasting plasma glucose, TC total cholesterol, TG triglyceride, LDL low density lipoprotein,

HDL High density lipoprotein, area under the ROC, CI Confidence interval

| **Supplementary Table 5 Relationship between four lipid-derived indicators and T2D in different models** | | | | |
| --- | --- | --- | --- | --- |
|  | **HR (95% CI)** | | | |
|  | **Model Ⅰ** | **Model Ⅱ** | **Model Ⅲ** | **Model Ⅳ** |
| **TG/HDL** | 1.24 (1.21, 1.27) | 1.17 (1.14, 1.2) | 1.12 (1.09, 1.15) | - |
| **TG/HDL Quintiles** |  |  |  |  |
| Q1 | Reference | Reference | Reference | - |
| Q2 | 1.52 (1.24, 1.87) | 1.34 (1.09, 1.65) | 1.37 (1.12, 1.68) | - |
| Q3 | 1.98 (1.63, 2.4) | 1.56 (1.29, 1.9) | 1.55 (1.28, 1.88) | - |
| Q4 | 2.84 (2.36, 3.42) | 2.04 (1.69, 2.47) | 1.94 (1.61, 2.34) | - |
| Q5 | 3.84 (3.21, 4.61) | 2.53 (2.1, 3.04) | 2.2 (1.83, 2.64) | - |
| P for trend | < 0.001 | < 0.001 | < 0.001 | - |
| **LDL/HDL** | 1.06 (1.01, 1.12) | 0.94 (0.88, 0.99) | 0.92 (0.87, 0.98) | 1.1 (1.04, 1.15) |
| **LDL/HDL Quintiles** |  |  |  |  |
| Q1 | Reference | Reference | Reference | Reference |
| Q2 | 1.17 (1, 1.36) | 1.06 (0.91, 1.23) | 1.02 (0.87, 1.19) | 1.27 (1.08, 1.48) |
| Q3 | 1.33 (1.15, 1.54) | 1.12 (0.96, 1.29) | 1.06 (0.92, 1.23) | 1.43 (1.23, 1.65) |
| Q4 | 1.37 (1.19, 1.58) | 1.08 (0.94, 1.25) | 1.01 (0.87, 1.16) | 1.46 (1.26, 1.68) |
| Q5 | 1.33 (1.15, 1.54) | 0.96 (0.83, 1.11) | 0.96 (0.83, 1.11) | 1.43 (1.24, 1.65) |
| P for trend | < 0.001 | 0.378 | 0.379 | < 0.001 |
| **Non-HDL/HDL** | 1.07 (1.03, 1.11) | 0.95 (0.91, 0.99) | 0.93 (0.89, 0.97) | 0.93 (0.89, 0.97) |
| **Non-HDL/HDL Quintiles** |  |  |  |  |
| Q1 | Reference | Reference | Reference | Reference |
| Q2 | 1.81 (1.53, 2.14) | 1.27 (1.07, 1.5) | 1.14 (0.97, 1.35) | 1.34 (1.14, 1.59) |
| Q3 | 2.28 (1.94, 2.67) | 1.23 (1.04, 1.44) | 1.08 (0.92, 1.27) | 1.26 (1.07, 1.49) |
| Q4 | 2.64 (2.27, 3.07) | 1.13 (0.96, 1.33) | 1.03 (0.88, 1.21) | 1.17 (1, 1.38) |
| Q5 | 3.15 (2.72, 3.64) | 1.01 (0.85, 1.19) | 0.88 (0.74, 1.03) | 1 (0.85, 1.18) |
| P for trend | < 0.001 | 0.055 | 0.001 | 0.02 |
| TyG | 4.98 (4.63, 5.35) | 4.36 (4.05, 4.7) | - | - |
| TyG Quintiles |  |  |  |  |
| Q1 | Reference | Reference | - | - |
| Q2 | 2.02 (1.55, 2.62) | 1.84 (1.41, 2.39) | - | - |
| Q3 | 3.02 (2.35, 3.89) | 2.55 (1.99, 3.28) | - | - |
| Q4 | 6.95 (5.47, 8.83) | 5.44 (4.27, 6.91) | - | - |
| Q5 | 15.97 (12.56, 20.31) | 11.52 (9.04, 14.67) | - | - |
| P for trend | < 0.001 | < 0.001 | - | - |

Model I: adjusted for age, gender, systolic blood pressure, diastolic blood pressure, alanine aminotransferase, blood urea nitrogen, serum creatinine, smoking status, drinking status and family history of diabetes. TG/HDL adjusted model I + TC + LDL; LDL/HDL adjusted model I + TC; Non-HDL/HDL adjusted model I + TC+ LDL; TyG adjusted model I + TC + LDL + HDL

Model II: adjusted for model and BMI

Model III: adjusted for model and FPG

Model Ⅳ: adjusted for model and TG

BMI body mass index, TyG triglyceride glucose index, FPG fasting plasma glucose, TC total cholesterol, TG triglyceride, LDL low density lipoprotein, HDL High density lipoprotein, HR hazard ratio, CI Confidence interval

| **Supplementary table 6 Subgroup analysis of four lipid-derived indicators** | | | | | | | | | | | |
| --- | --- | --- | --- | --- | --- | --- | --- | --- | --- | --- | --- |
|  | **TG/HDL** | |  | **LDL/HDL** | |  | **Non-HDL/HDL** | |  | **TyG** | |
|  | HR (95% CI) | *P*_intreaction_ |  | HR (95% CI) | *P*_intreaction_ |  | HR (95% CI) | *P*_intreaction_ |  | HR (95% CI) | *P*_intreaction_ |
| **BMI group** |  | 0.803 |  |  | 0.001 |  |  | < 0.001 |  |  | 0.877 |
| <18.5 kg/m^2^ | 1.07 (0.55, 2.07) |  |  | 1.17 (0.57, 2.42) |  |  | 1.07 (0.59, 1.93) |  |  | 2.96 (0.91, 9.68) |  |
| 18.5-24 kg/m^2^ | 1.33 (1.26, 1.41) |  |  | 1.09 (0.99, 1.2) |  |  | 1.09 (1.04, 1.15) |  |  | 5.63 (4.86, 6.51) |  |
| 24-28 kg/m^2^ | 1.19 (1.14, 1.24) |  |  | 0.9 (0.83, 0.99) |  |  | 0.94 (0.88, 1) |  |  | 4.25 (3.82, 4.72) |  |
| ≥28 kg/m^2^ | 1.09 (1.03, 1.15) |  |  | 0.87 (0.77, 0.98) |  |  | 0.87 (0.81, 0.95) |  |  | 3.51 (3.02, 4.08) |  |
| **FPG Tertiles** |  | 0.116 |  |  | 0.03 |  |  | 0.001 |  |  | - |
| Low | 1.18 (1.05, 1.33) |  |  | 1.11 (0.85, 1.43) |  |  | 1.18 (1, 1.4) |  |  | - |  |
| Middle | 1.26 (1.15, 1.38) |  |  | 1.14 (0.99, 1.32) |  |  | 1.13 (1.03, 1.23) |  |  | - |  |
| High | 1.16 (1.13, 1.19) |  |  | 0.93 (0.88, 0.99) |  |  | 0.96 (0.91, 1) |  |  | - |  |
| **TG Tertiles** |  | - |  |  | 0.001 |  |  | 0.047 |  |  | - |
| Low | - |  |  | 1.25 (1.02, 1.52) |  |  | 0.76 (0.61, 0.96) |  |  | - |  |
| Middle | - |  |  | 1.06 (0.95, 1.17) |  |  | 0.92 (0.83, 1.03) |  |  | - |  |
| High | - |  |  | 0.85 (0.79, 0.92) |  |  | 0.89 (0.84, 0.94) |  |  | - |  |
| All models were adjusted for age, gender, systolic blood pressure, diastolic blood pressure, alanine aminotransferase, blood urea nitrogen, serum creatinine, smoking status, drinking status and family history of diabetes.  BMI body mass index, TyG triglyceride glucose index, FPG fasting plasma glucose, TC total cholesterol, TG triglyceride, LDL low density lipoprotein, HDL High density lipoprotein, HR hazard ratio, CI Confidence interval | | | | | | | | | | | |
